# Area socioeconomic status is independently associated with esophageal cancer mortality in Shandong, China

**DOI:** 10.1038/s41598-019-42774-x

**Published:** 2019-04-23

**Authors:** Kou Kou, Peter David Baade, Xiaolei Guo, Michelle Gatton, Susanna Cramb, Zilong Lu, Zhentao Fu, Jie Chu, Aiqiang Xu, Jiandong Sun

**Affiliations:** 1 School of Public Health and Social Work, Queensland University of Technology, Brisbane, Australia; 2 Cancer Research Centre, Cancer Council Queensland, Brisbane, Australia; 3 Shandong Centre for Disease Control and Prevention, Jinan, China

**Keywords:** Cancer epidemiology, Risk factors

## Abstract

Esophageal cancer (EC) is a leading cause of cancer death in China. Within Shandong Province, a geographic cluster with high EC mortality has been identified, however little is known about how area-level socioeconomic status (SES) is associated with EC mortality in this province. Multilevel models were applied to EC mortality data in 2011–13 among Shandong residents aged 40+ years. Area-level SES factors consisted of residential type (urban/rural) of the sub-county-level units (n = 262) and SES index (range: 0–10) of the county-level units (n = 142). After adjustment for age and sex, residents living in rural areas had a 22% (95% CI: 13–32%) higher risk of dying from EC than those in urban areas. With each unit increase in the SES index, the average risk of dying from EC reduced by 10% (95% CI: 3–18%). The adjustment of area-level SES variables had little impact on the risk ratio of EC mortality between the high-mortality cluster and the rest of Shandong. In conclusion, rural residence and lower SES index are strongly associated with elevated risks of EC death. However, these factors are independent of the high mortality in the cluster area of Shandong. The underlying causes for this geographic disparity need to be further investigated.

## Introduction

**Figure 1:**
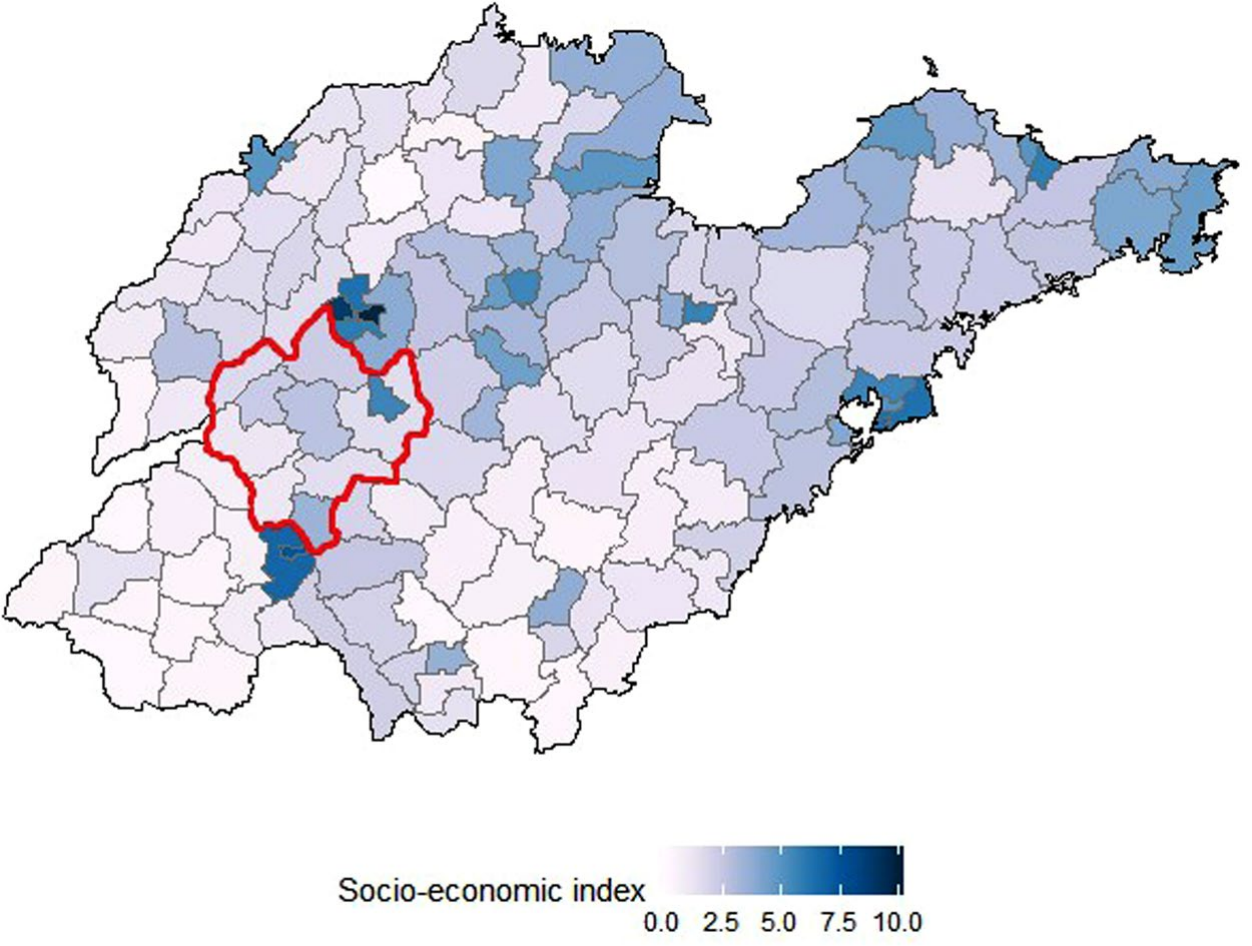
Distribution of county-level SES index over Shandong, China, 2011–2013. *Note:* *The color shows the scores of socio-economic index for the 142 counties in Shandong Province.* *Area with red outline is high-mortality cluster identified by previous study*^3^.

Esophageal cancer (EC) is an important public health issue in China. It is the fourth leading cause of cancer mortality, causing 197,800 deaths in China in 2013, which accounted for nearly half of the total EC deaths worldwide^1,2^. The age-standardized mortality rate (ASMR) ranges almost ten-fold (4.6 to 42.5 per 100,000 population) across different provinces in China^1^. There are also large intra-provincial variations in EC mortality. For example, within the Shandong Province of China, the EC mortality in the eastern region of the province was lower than in the central and western regions, with a consistently high-mortality risk cluster identified in the mid-west region (Fig. 1). Residents living in the cluster area were nearly four times more likely to die from EC than residents in the rest of Shandong (RR: 3.7, 95% CI: 2.8–5.0)^3^.

Studies have shown that lower socioeconomic status (SES) is associated with increased incidence or mortality due to EC^4–10^. In the Chinese population, the majority of the studies have been cohort or cross-sectional studies focused on an individual’s SES^8–10^. Population-based studies considering area-level effects are scarce in China. Hence, they are not able to indicate whether, and to what extent, the geographic disparities of EC mortality are due to regional resources.

In Shandong, the economy in the eastern region is more developed compared to the central and western regions^11^. Given the consistency of this economic distribution with the above-mentioned patterns in EC mortality in Shandong, we hypothesize that the area SES is associated with the geographic disparity in EC morality in Shandong population.

In this study, we build on and extend previous studies by using multilevel analysis to simultaneously account for variation in EC mortality across areas and between individuals in Shandong population, accounting for 7.2% of the Chinese population^12^. The analysis aims to elucidate potential associations between area-level SES factors and EC mortality among the Shandong population, thus furthering the understanding of how place matters to cancer outcome. Specifically, we aimed to examine the relationships between residential type (sub-county level), SES index (county-level) and EC mortality, and explore whether these area-level SES factors explain the previously identified high-mortality cluster in Shandong.

## Results

### SES index

Estimates of county-level SES index (range 0 to 10) were generated based on the average GDP per capita, average years of school education, and number of hospital beds per capita of the 142 county-level units in Shandong. Across all 142 counties, the median SES index was 2.1. The geographic distribution of the SES index in Shandong is shown in Fig. 1. The SES indexes of counties located in cluster areas were not significantly different from the rest counties in Shandong based on Mann-Whitney U test (p = 0.962). The 142 counties-level units were classified into 97 low SES counties, 39 middle SES counties, and 6 high SES counties based on their SES index values for descriptive analysis.

### Study population

Selected characteristics of the study population are summarized in Table 1. In 2011–2013, a total of 45,646 EC deaths were reported, with a crude mortality rate of 33.6 per 100,000 person-years among people aged 40+ years. The ASMR was 36.0 (95% CI: 30.6–42.3) per 100,000 person-years, using the 2013 Global Burden of Disease population^13^. For reporting purposes, age groups were aggregated into 10-year age groups. The ASMR increased with age (p < 0.001), from 3.3 (95% CI: 1.3–7.0) in people aged 40–49 to 151.2 (95% CI: 104.5–212.6) in people aged 80+ years. Across all ages, males had 3.3 times higher ASMR than females (56.4 vs 17.2; p < 0.01).

**Table 1:** Cohort description and EC mortality outcomes in Shandong, 2011–2013.

	EC death (N)	Person-years (N)	ASMR^*^ (95% CI)	p^§^
***Individual-level variables***				
**Age at death (p** < **0.001)**				
40–49	1,715	51,701,730	3.3 (1.3–7.0)	—
50–59	7,001	38,321,236	17.8 (11.7–26.1)	<0.001
60–69	13,202	25,766,507	51.7 (38.4–68.3)	<0.001
70–79	14,787	14,095,364	104.3 (78.6–134.9)	<0.001
80+	8,941	5,867,644	151.2 (104.5–212.6)	<0.001
**Sex (p** < **0.001)**				
Female	11,135	68,261,387	17.2 (13.6–21.5)	
Male	34,511	67,491,094	56.4 (49.4–64.5)	<0.001
***Sub-county-level variable***				—
**Residential type (p** < **0.001)**				
Urban	12,346	45,478,561	29.4 (23.5–36.7)	—
Rural	33,300	90,273,920	39.3 (34.4–44.9)	0.001
***County-level variables***				
**GDP per capita (CHY**^**†**^ **1000 Yuan) (p** = **0.564)**				
High (95.7–138.2)	508	2,216,660	24.2 (18.5–31.2)	—
Middle (53.1–95.6)	4,937	21,525,902	24.0 (19.2–29.8)	—
Low (10.4–53.0)	40,201	112,009,919	38.7 (33.2–44.9)	—
**Average school education (Years) (p** = **0.001)**				
High (11.1–12.8)	1,688	8,893,539	20.2 (15.6–26.0)	—
Middle (9.3–11.0)	5,051	17,562,265	31.6 (25.6–38.8)	0.008
Low (7.4–9.2)	38,907	109,296,677	38.1 (32.7–44.1)	<0.001
**Number of hospital beds (per 1000 persons) (p** = **0.115)**				
High (16.0–23.1)	607	2,735,505	23.0 (18.0–29.1)	
Middle (8.9–15.9)	2,544	11,321,489	24.4 (19.4–30.4)	
Low (1.7–8.8)	42,495	121,695,487	37.4 (32.0–43.6)	
**SES index** **(p < 0.001)**				
High (most advantaged areas)	831	4,375,789	19.5 (15.1–24.8)	—
Middle	6,772	27,871,478	26.1 (20.8–32.4)	<0.001
Low (least advantaged areas)	38,043	103,505,214	39.5 (34.1–45.6)	<0.001

^*^ASMR: Age-standardized mortality rate, per 100,000 person-years.

^§^p-values were calculated using negative binomial models adjusted for age and the corresponding variable. p-values for comparisons between categories are not presented if the variables are not significant. Variables’ p-values are the p-values for linear combinations of model coefficients.

^†^CHY: Chinese Yuan.

### Area-level SES and EC mortality

**Figure 2:**
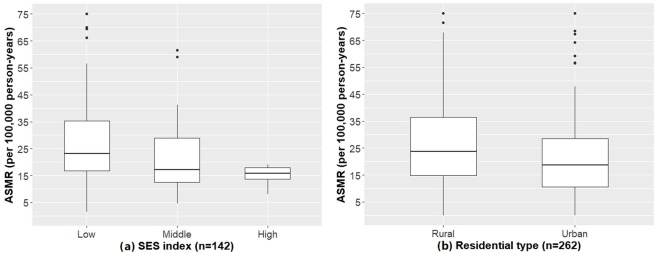
Mortality rate of EC by different levels of SES variables in Shandong, China, 2011–2013. *Note:* (**a**) *Was based on the ASMRs of 142 county-level units, 5 units with ASMR more than 75 were truncated to 75 per 100,000 person-years;* (b) *was based on the ASMRs of 262 sub-county-level units, 11 units with ASMR more than 75 were truncated to 75 per 100,000 person-years*. *The SES index ranged from 0 to 10, with low SES index from 0.0 to 3.3, middle SES index from 3.4 to 6.7, and high SES index from 6.8 to 10.0*.

The ASMR of the 142 counties ranged from 1.5 to 144.0 per 100,000 person-years. ASMR was higher in rural areas than urban areas (39.3 vs 29.4; p = 0.001); and the highest ASMRs were seen in counties with low SES index (p < 0.001) and low average years of school education (p < 0.001) (Table 1). A gradient of increasing ASMR was observed as the SES index decreased, or residential type changed from urban to rural (Fig. 2).

In the cluster area, a total of 10,249 deaths were reported during 2011–2013 out of 10,362,408 person-years; In the rest of Shandong, 35,397 deaths were reported out of 125,390,073 person-years. The crude risk ratio for EC mortality between cluster and non-cluster area is 3.39 (95% CI: 2.25–5.11; p < 0.001). After adjusting for age and sex, the risk ratio increased to 3.84 (95% CI: 2.59–5.70; p < 0.001).

Similar to the whole Shandong population, the ASMR increased with age and was higher in males in both cluster (p < 0.001) and non-cluster areas (p < 0.001) (Table 2). Also, in both areas, rural residents were more likely to die from EC than urban residents (p = 0.026 in cluster areas, p = 0.023 in non-cluster areas); and the ASMR increased with the decrease of county-level SES index in cluster area (p < 0.001) and the trend is close to being significant in non-cluster areas (p = 0.055).

**Table 2:** Mortality by demographic and socioeconomic factors in cluster and non-cluster areas^*^ of Shandong, 2011–2013.

Demographic/SES factors	Cluster		Non-cluster	
	ASMR^†^ (95% CI)	p^§^	ASMR (95% CI)	p^§^
Overall	108.1 (98.8–118.2)	—	30.3 (25.3–36.0)	—
***Individual-level variables***				
**Age**		<**0.001**		<**0.001**
40–49	11.0 (6.9–16.6)	—	2.7 (0.9–6.4)	—
50–59	61.5 (49.6–75.5)	<0.001	14.2 (8.6–22.2)	<0.001
60–69	157.8 (133.9–184.9)	<0.001	42.8 (30.3–58.9)	<0.001
70–79	308.7 (261.8–361.7)	<0.001	88.5 (64.4–118.8)	<0.001
80+	430.4 (344.9–532.0)	<0.001	130.3 (85.8–191.0)	<0.001
**Sex**		<**0.001**		<**0.001**
Female	60.9 (54.1–68.3)	—	13.6 (10.4–17.6)	—
Male	160.0 (148.1–172.9)	<0.001	48.2 (41.7–55.8)	<0.001
***Sub-county-level variables***				
**Residency**		**0.026**		**0.023**
Urban	77.2 (67.1–88.7)	—	26.0 (20.4–32.8)	—
Rural	121.3 (113.0–130.2)	0.026	32.4 (27.9–37.5)	0.023
***County-level variables***				
**SES index**		<**0.001**		**0.055**
High (most advantaged areas)	—	—	19.5 (15.1–24.8)	—
Middle	48.6 (42.3–55.9)	—	24.4 (19.3–30.7)	—
Low (least advantaged areas)	121.7 (111.9–132.3)	<0.001	32.4 (27.5–37.9)	—

^*^Cluster area is a region with consistently high-mortality risk of EC in Shandong Province3 (Fig. 1). Non-cluster areas are the rest of the province.

^†^ASMR: Age-standardized mortality rate, per 100,000 person-years.

^§^p-values were calculated using negative binomial models adjusted for age and the corresponding variable. p-values for comparisons between categories are not presented if the variables are not significant. Variables’ p-values are the p-values for linear combinations of model coefficients.

### Multilevel regression

The multilevel model revealed the association between area SES and EC mortality in the Shandong population (Table 3, Model 1). After adjustment for age and sex, residents living in rural areas had 22% (95% CI: 13–32%; p < 0.001) higher risk of dying from EC. With each point increase of SES index in a county, the average risk of dying from EC reduced by 10% (95% CI: 3–18%; p = 0.010) for the residents living that county.

**Table 3:** Modelling the association between residential type and cancer mortality.

	Model 1			Model 2		
	Relative risk	95% CI	p	Relative risk	95% CI	p
***Individual-level variables***						
**Age group**						
40–49	1	—		1	—	
50–59	5.20	4.80–5.63	<0.001	5.20	4.80–5.63	<0.001
60–69	15.74	14.57–17.01	<0.001	15.73	14.56–17.00	<0.001
70–79	36.77	34.03–39.73	<0.001	36.76	34.02–39.73	<0.001
80+	63.17	58.37–68.37	<0.001	63.16	58.34–68.37	<0.001
**Sex**						
Female	1	—		1	—	
Male	4.39	4.08–4.71	<0.001	4.38	4.09–4.70	<0.001
***Sub-county-level variable***						
**Residential type**						
Urban	1	—		1	—	
Rural	1.22	1.13–1.32	<0.001	1.22	1.13–1.32	<0.001
***County-level variables***						
SES index^*^	0.90	0.82–0.97	0.010	0.90	0.84–0.97	0.008
**Cluster variable**						
Non-cluster	—	—	—	1	—	
Cluster	—	—	—	3.80	2.56–5.65	<0.001

*SES index is continuous variable here to give the best model fit.

The interaction between residential type and SES index on EC mortality was not statistically significant (RR: 1.01; 95% CI: 0.95–1.06; p = 0.84), supporting the hypothesis that the influence of residential type and SES index with EC mortality were independent of each other.

Model 2 elucidated to what extent the area-level SES variables explain the identified high-mortality cluster (Table 3, Model 2). After adjustment for age and sex, the risk ratio of EC mortality between cluster and non-cluster area was 3.84 (95% CI: 2.59–5.70; p < 0.001). After additional adjustment for area-level SES, this reduced slightly to 3.80 (95% CI: 2.56–5.65; p < 0.001).

Interactions between the cluster variable and residential type (RR: 1.11; 95% CI: 0.85–1.44; p = 0.46), and cluster variable and SES index (RR: 0.72; 95% CI: 0.49–1.08; p = 0.11) were not significant, suggesting that the relationship between area-level SES and EC mortality did not differ between cluster and non-cluster areas.

**Figure 3:**
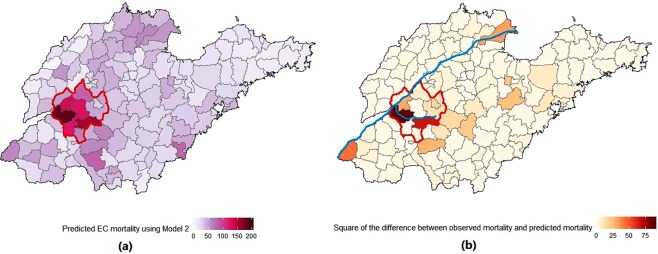
Predicted county-level EC mortality and difference between observed and predicted mortality in Shandong, China, 2011–2013. *Note:* (**a**) *is predicted mortality using Model 2* (*in per 100,000 person-years*) (**b**) *is squared difference between observed mortality and predicted mortality. Area with red outlines are high-mortality cluster. Blue lines represent Yellow River* (*upper line*) *and its branch Dawen river* (*lower line*).

The differences between observed EC mortality and predicted EC mortality (using Model 2) of the 142 counties have been visualized on the Shandong map (Fig. 3). The biggest differences were seen in two counties (Dongping and Ningyang) located in the cluster area. The predicted EC mortality of these two counties (184.0 per 100,000 person-year in Dongping and 155.6 in Ningyang) based on Model 2 is higher than the observed mortality (175.7 and 148.5, respectively). The urban to rural population ratio was 0.53 in Dongping and 0.14 in Ningyang compared to 0.55 in Shandong. The SES index were 1.2 and 1.5, respectively in these two counties compared to the median SES index of 2.1 for the 142 counties.

## Discussion

This multilevel study shows that EC mortality is not only associated with an individual’s characteristics, such as age and gender, but also depends on the SES of where people live.

People living in rural areas had a 22% higher risk of dying from EC compared with the urban residents. This pattern was independent from age, sex, and the SES index. There are several possible reasons for the observed association between residential type and EC mortality. First, the prevalence of modifiable risk factors of EC might be higher among rural residents. For example, a recent study reported that people in rural areas of China are more likely to smoke and drink than their urban counterparts^14^. The onset of EC can be associated with Human Papillomavirus, which are more prevalent in rural than urban populations^15,16^. Since the survival rate of EC is relatively low^17^, higher incidence in rural areas is likely to translate to a higher mortality.

Second, disparities in health services between rural and urban areas may result in delayed diagnosis in rural patients which leads to poor survival. In China, the majority of the tertiary referral hospitals are in urban areas. The different health insurance system between urban and rural residents leads to longer wait times for medical investigation and referral for diagnosis for rural patients^18,19^. A Chinese study has shown that breast cancer patients from rural areas were significantly more likely than urban patients to present with late-stage diseases^20^. EC patients from rural areas might have the same situation.

Furthermore, financial disadvantage may also lead to lower survival among rural patients and increase mortality. Rural residents on average have less income than urban residents^21^. Because of the different health insurance system, rural patients may need to pay more out-of-pocket expenses for treating their illness even when admitted to the same hospitals^18^. This might lead to delayed or suboptimal treatment among the rural cancer patients^20^.

We developed a summary measure of county-level SES (SES index) which incorporates GDP per capita, average years of school education, and number of hospital bed per capita. The results illustrate that average EC mortality of a county is associated with the county’s SES index. The results reflect the results from a Chinese national study, which found the GDP per capita at a county level was inversely associated with EC incidence and mortality^22^.

GDP per capita is a useful measurement when comparing differences in living standards between different regions. It also reflects inequalities in EC risk factors such as smoking, and alcohol use^23^. In addition, local governments in affluent areas with a high GDP level are more likely to allocate a larger proportion of the total expenditure on health care, which may lead to better access to health resources^24^. Our study also found a significant correlation between a county’s GDP per capita and number of hospital bed per capita. As a result, a better prognosis and a lower EC mortality could be achieved in areas with higher GDP per capita.

Residents with lower education level were related with suboptimal nutrition and higher prevalence of smoking in the Chinese population^8,25^, which could contribute to a high EC incidence and mortality. A study about breast cancer reported that, in China, lower education level was related to later cancer staging at diagnosis and lower success rates of cancer treatment, therefore leads to worse survival^26^. Our study adds to the existing knowledge by showing a direct link between poor education and increased EC mortality at a population level.

In a previous study we identified a cluster with higher EC risk in Shandong Province. The cluster is located in the mid-west region of Shandong, which has been reported as a less developed region^11^. Our initial hypothesis was the geographic disparity of EC mortality would be associated with the inequitable area-level SES. However, the risk ratio of area-level SES variables was not influenced by including the cluster variable (Model 1 vs Model 2). This suggests these area-level SES factors are highly unlikely to have anything to do with the large difference in EC mortality between the high- and low-risk regions in Shadong, despite their strong associations with the overall mortality.

The biggest differences between observed mortaltiy and predicted mortality were seen in two counties located in the cluster area (Fig. 3). The residential type proportion and SES index of these two counties were not different from other counties in Shandong, suggesting that our current model, which adjusted for age, sex, area-level SES factors, and cluster variable, could not fully explain the EC mortality in these two counties. Interestingly, both of these counties are in the downstream catchment area of the Dawen River. Future investigation especially regarding water influences are needed to elucidate the factors underlying this high EC mortality area in China.

This study has several limitations. First, our analyses were based on the total EC mortality with no information on histological types. EC mainly consists of two different histological types (squamous cell carcinoma (SCC) and adenocarcinoma) with different epidemiologic features. The main risk factors of SCC are alcohol and tobacco use^27^, which are more prevalent in socio-economic disadvantaged areas in China. In contrast, the main risk factors of adenocarcinoma include obesity^27^, which is more common in advantaged areas^28^. Thus, the relationship between EC mortality and SES would likely be different between those two histologic types. Research data have shown that more than 90% of EC cases in China are SCC^29^. Therefore, the small numbers of adenocarcinoma cases might not significantly influence the association between SES and SCC cases in this study. In addition, this study did not include many individual-level characteristics. As the income inequality is relatively high in China^30^, an individual’s SES may be different from the county-level SES index. Also, the SES index could be different between urban and rural sub-county units. Adjusting for individual SES variables such as occupation and income in the model or including sub-county-level SES index would help address this limitation.

## Conclusion

In this multilevel study of EC mortality with a large Chinese population, we found that area-level SES factors were independently associated with EC mortality. However, these factors seem to be independent of the large difference in EC mortality between the low- and high-risk regions. The underlying causes for the observed geographic disparity need to be further investigated to inform public health policies and programs to lower the high disease burden and to address inequalities.

## Methods

### Study site and units of analysis

Shandong Province is a coastal province in North-East China, covering an area of 157,100 square kilometres. It is the second most populous province in China, with about 99% of its 98.5 million people belonging to the Han ethnic group^31^.

Variation in EC mortality was analyzed according to the hierarchical structure where the numbers of death by age and sex (individual-level) were nested within sub-county-level units (sub-county-level) within county-level units (county-level). For this study, we divided the total Shandong population (without gaps or overlaps^32^) into 142 county-level units according to gazetted administrative boundaries. Counties were further subdivided into 262 sub-county-level units according to residential type.

### Cancer data sources

Deaths caused by EC (ICD-10: C15, malignant neoplasm of esophagus) in the population aged 40+ years residing in Shandong Province between 1^st^ January 2011 and 31^st^ December 2013 were extracted from the Shandong Death Registration System (SDRS) database. EC-specific death information was collected based on a national standard protocol^33^ and were double checked by physicians and their direct managers. More details about the quality of the data has been discussed elsewhere^3,11,32^. The extracted de-identified EC death dataset represents the number of EC deaths in each of the 262 sub-county-level units in Shandong by sex, and 5-year age group. The age groups above 85 years were combined into an 85+ age group.

The population for each sub-county-level unit by the same age-sex categories were obtained from Shandong Centre for Disease Control and Prevention (Shandong CDC), who generated their estimates based on data from local police departments and statistical bureaus^31^.

### Measurement of sub-county-level SES

Residential type is the sub-county-level SES variable and consists of two levels: urban and rural. Sub-county-level units consisting of townships are classified as rural areas. A township is an administrative unit consisting of a town center and dozens of surrounding villages. Sub-county-level units made up of subdistricts are classified as urban areas. A subdistrict consists of urban communities or neighborhoods clustering around the center of a county/city/district. More information about the residential type classification in Shandong has been published previously^32^.

Residential type has been reported as a strong indicator for an individual’s income and socioeconomic class in China, as rural residents on average have less household income per head, less mean years of education, less bank savings, and a higher percentage of blue collar workers than urban residents^21,34,35^.

### Measurement of county-level SES

The county-level (n = 142) SES variables considered were average gross domestic product (GDP) per capita, average years of school education for adults, and number of hospital beds per capita. The 2011–2013 data for these measurement were extracted from the Shandong Statistical Yearbooks^31^.

Pearson’s correlation suggested significant correlations between the three county-level SES variables, with the coefficient between GDP and education being 0.5 (p < 0.001); GDP and number of hospital beds being 0.3 (p < 0.001); and number of hospital beds and education years being 0.7 (p < 0.001). Therefore, rather than treating these measures separately, principal component analysis (PCA) was used to generate an SES index. The benefits and applications of constructing an SES index with PCA has been published before^36–38^. In this study, components with an eigenvalue of 1.0 or higher was chosen as SES index.

Three components were created from the county-level SES variables. Only one of the components had an eigenvalue more than 1 (eigenvalue = 2.00), explaining 66.7% of variation in the original SES variables. This component was transformed to scale from 0 to 10 using the min-max normalization method:

$${x}^{\ast }=\frac{x-{x}_{\min }}{{x}_{\max }-{x}_{\min }}\times 10$$

where x* is the transformed value, *x* is the initial value of the component, *x*_*min*_ is the minimum value of the component, *x*_*max*_ is the maximum value of the component. The transformed component x* was used as the SES index in this study, with the score of 0 representing the least advantaged area and 10 representing the most advantaged area.

For descriptive purposes in Tables 1 and 2, the continuous SES index was categorized into three groups based on its value (low SES index from 0.0 to 3.3, middle from 3.4 to 6.7, and high from 6.8 to 10.0). In multilevel regression model (Table 3), we used the continuous SES index variable directly.

### Multilevel regression

Multilevel mixed effect negative binomial models were used to examine the association between area-level SES (i.e. residential type and SES index) and EC mortality (Model 1), and to estimate how area-level SES factors contribute to the high-mortality cluster in Shandong (Model 2).

Specifically, Model 1 concurrently included age, sex, residential type, and SES index to estimate the independent contribution of the two area-level SES factors on EC mortality. Interaction between residential type and SES index was also considered to ascertain if the relationship between county-level SES index and EC mortality differed by residential type.

Model 2 added a cluster variable (cluster/non-cluster) to Model 1, to elucidate the contribution of area SES factors on the mortality ratio between cluster and non-cluster areas. Interactions between the cluster variable and residential type, cluster variable and SES index were considered to clarify if the contribution of area SES on EC mortality differed between cluster and non-cluster areas.

Data were analyzed and visualized using R software (Version 3.4.3) and Stata (version 15.0). Parameter estimates are presented as risk ratios (RR) with their 95% confidence intervals (CI).

### Ethical approval

The study protocols were approved by the Shandong CDC Ethics Committee (No. 2013–20) and have obtained the confirmation of human research ethics exemption from Queensland University of Technology Research Ethics Advisory Team (Exemption No. 1600000838).

## Data Availability

The data that support the findings of this study are available from Shandong Center for Disease Control and Prevention, but restrictions apply to the availability of these data, which were used under license for the current study, and so are not publicly available. Data are however available from the authors upon reasonable request and with permission of Shandong Center for Disease Control and Prevention.

## References

[1] Zhou M (2016). Cause-specific mortality for 240 causes in China during 1990-2013: a systematic subnational analysis for the Global Burden of Disease Study 2013. Lancet.

[2] Fitzmaurice C (2017). Global, regional, and national cancer incidence, mortality, years of life lost, years lived with disability, and disability-adjusted life-years for 32 cancer groups, 1990 to 2015: a systematic analysis for the global burden of disease study. JAMA Oncol..

[3] Kou K (2018). Spatial analysis of esophageal cancer mortality in a high-risk population in China: consistent clustering pattern in 1970–74 and 2011–13. Asian Pac. J. Cancer Prev..

[4] LeVea, C. Pathogenesis of esophageal cancer in *Minimally invasive foregut surgery for malignancy* (ed. Hochwald, S. N., Kukar, M.) 1–9 (Springer, 2015).

[5] Mohebbi M (2011). The spatial distribution of esophageal and gastric cancer in Caspian region of Iran: An ecological analysis of diet and socio-economic influences. Int. J. Health Geogr..

[6] Islami F (2009). Socio-economic status and oesophageal cancer: results from a population-based case-control study in a high-risk area. Int. J. Epidemiol..

[7] Brown LM (2001). Excess incidence of squamous cell esophageal cancer among US Black men: role of social class and other risk factors. Am. J. Epidemiol..

[8] Tran GD (2005). Prospective study of risk factors for esophageal and gastric cancers in the Linxian general population trial cohort in China. Int. J. Cancer..

[9] Yu Y (1993). Retrospective cohort study of risk-factors for esophageal cancer in Linxian, People’s Republic of China. Cancer Cause Control.

[10] Xibin S (2003). Risk factors for oesophageal cancer in Linzhou, China: a case-control study. Asian Pac. J. Cancer Prev. P.

[11] Chu J (2017). Female breast cancer mortality clusters in Shandong Province, China: a spatial analysis. Sci. Rep..

[12] Ma, J. *et al*. *Tabulation on the 2010 population census of the People’s Republic of China*. (China Statistics Press, 2012).

[13] Naghavi M (2015). Global, regional, and national age-sex specific all-cause and cause-specific mortality for 240 causes of death, 1990-2013: a systematic analysis for the Global Burden of Disease Study 2013. Lancet.

[14] Liao Y, Chen X, Tang J (2017). Differences of cigarette smoking and alcohol consumption by sex and region in China: a population-based, multi-stage, cluster sampling survey. Lancet.

[15] Zahnd WE (2017). Rural-Urban differences in cancer incidence and trends in the United States. Cancer Epidemiol. Biomark. Prev..

[16] Zhang SK (2015). The association between human papillomavirus 16 and esophageal cancer in Chinese population: a meta-analysis. BMC cancer.

[17] Rustgi AK, El-Serag HB (2014). Esophageal carcinoma. N. Engl. J. Med..

[18] Klotzbücher S, Lässig P, Qin J, Weigelin-Schwiedrzik S (2010). What is new in the “New Rural Co-operative Medical System”? An assessment in one Kazak county of the Xinjiang Uyghur autonomous region. China Q..

[19] Liu M, Zhang Q, Lu M, Kwon CS, Quan H (2007). Rural and urban disparity in health services utilization in China. Med. Care.

[20] Peng Z (2016). Diagnosis and treatment pattern among rural and urban breast cancer patients in Southwest China from 2005 to 2009. Oncotarget.

[21] Sicular T, Ximing Y, Gustafsson B, Shi L (2007). The urban–rural income gap and inequality in China. Rev. Income Wealth.

[22] Yang Z (2017). Comparison of cancer incidence and mortality in three GDP per capita levels in China, 2013. Chin. J. Cancer Res..

[23] Stuckler D, McKee M, Ebrahim S, Basu S (2012). manufacturing epidemics: the role of global producers in increased consumption of unhealthy commodities including processed foods, alcohol, and tobacco. Plos Med..

[24] Pritchard, C., Hickish, T. Changes in cancer incidence and mortality in England and Wales and a comparison of cancer deaths in the major developed countries by age and sex 1979–2002 in context of GDP expenditure on health. *ecancermedicalscience* **2** (2008). 10.3332/eCMS.2008.80 PMC3234042 22275969

[25] Li Q, Hsia J, Yang G (2011). Prevalence of smoking in China in 2010. N. Engl. J. Med..

[26] Liu, Y. *et al*. Influence of occupation and education level on breast cancer stage at diagnosis, and treatment options in China: A nationwide, multicenter 10-year epidemiological study. *Medicine* **96** (2017). 10.1097/MD.0000000000006641 PMC5403113 28403116

[27] Pennathur A, Gibson MK, Jobe BA, Luketich JD (2013). Oesophageal carcinoma. Lancet.

[28] Zhang H (2017). Relation of socioeconomic status to overweight and obesity: a large population-based study of Chinese adults. Ann. Hum. Biol..

[29] Liang H, Fan JH, Qiao YL (2017). Epidemiology, etiology, and prevention of esophageal squamous cell carcinoma in China. Cancer Biology & Medicine.

[30] Xie Y, Zhou X (2014). Income inequality in today’s China. Proc. Natl. Acad. Sci. USA.

[31] National Bureau of Statistics of the People’s Republic of China. *Shandong Statistical Yearbook* (ed. Chen, D.) (China Statistics Press, 2015).

[32] Sun J (2018). The gap between cause-of-death statistics and Household Registration reports in Shandong, China during 2011-2013: Evaluation and adjustment for underreporting in the mortality data for 262 subcounty level populations. PloS One.

[33] National Health and Family Planning Commission of the People’s Republic of China. The standards of death registration *http://www.nhfpc.gov.cn/guihuaxxs/s10742/201401/38171e5c7bdf4526897da62a912a17f5.shtml* (2014).

[34] Zimmer Z, Kwong J (2004). Socioeconomic status and health among older adults in rural and urban China. J. Aging Health.

[35] Meng X, Zhang J (2001). The two-tier labor market in urban China: occupational segregation and wage differentials between urban residents and rural migrants in Shanghai. J. Comp. Econ..

[36] Vyas S, Kumaranayake L (2006). Constructing socio-economic status indices: how to use principal components analysis. health policy plann..

[37] Australian Bureau of Statistics. Socio-Economic Indexes for Areas, http://www.abs.gov.au/websitedbs/censushome.nsf/home/seifa (2018).

[38] Bentley R, Kavanagh AM, Subramanian S, Turrell G (2008). Area disadvantage, individual socio-economic position, and premature cancer mortality in Australia 1998 to 2000: a multilevel analysis. Cancer Causes Control.

